# Lipid Metabolism and Lipid Droplets in Pancreatic Cancer and Stellate Cells

**DOI:** 10.3390/cancers10010003

**Published:** 2017-12-23

**Authors:** Yoshiaki Sunami, Artur Rebelo, Jörg Kleeff

**Affiliations:** 1 Department of Visceral, Vascular and Endocrine Surgery, Halle University Hospital, Martin-Luther University Halle-Wittenberg, Halle 06120, Germany; artur.rebelo@uk-halle.de (A.R.); joerg.kleeff@uk-halle.de (J.K.); 2 Department of General, Visceral and Vascular Surgery, BG Hospital Bergmanstrost, Halle 06112, Germany

**Keywords:** pancreatic ductal adenocarcinoma, pancreatic stellate cells, lipid metabolism, lipid droplets, reprogramming in lipid metabolism

## Abstract

Pancreatic ductal adenocarcinoma (PDAC) is projected to become the second deadliest cancer by 2030, and the overall 5-year survival rate is currently less than 7%. Cancer cells frequently exhibit reprogramming of their metabolic activity. It is increasingly recognized that aberrant de novo lipid synthesis and reprogrammed lipid metabolism are both associated with the development and progression of various cancers, including pancreatic cancer. In this review, the current knowledge about lipid metabolism and lipid droplets in pancreatic cancer is discussed. In the first part, molecular mechanisms of lipid metabolism and roles of enzymes involved in lipid metabolism which are relevant for pancreatic cancer research are presented. Further, preclinical studies and clinical trials with drugs/inhibitors targeting cancer metabolic systems in cancer are summarized. An increase of our knowledge in lipid metabolism in pancreatic cancer cells and in tumor stroma is important for developing novel strategies of future individualized therapies of pancreatic cancer.

## 1. Introduction

Pancreatic ductal adenocarcinoma (PDAC) is a devastating disease with an unfavorable outcome due to late diagnosis, which is often due to a lack of sensitive and specific tumor markers despite enormous advances in our understanding of pancreatic cancer biology. In addition, pancreatic cancer exhibits remarkable resistance to established therapy options such as chemotherapy, radiotherapy, and molecularly targeted therapy. PDAC is projected to become the second deadliest cancer by 2030, and currently the overall 5-year survival rate is less than 7% [1,2]. To date, surgery remains the only curative option for pancreatic cancer. With increasing surgical experience, better patient-care and perioperative management, medical centers with high patient volume are able to conduct safe pancreatic cancer surgery. Macroscopic complete tumor resection and adjuvant therapies results in 5-year survival rates of more than 20% [2,3]. Nevertheless, more effective combinational chemotherapeutic options, better preoperative treatments, and postoperative adjuvant therapy could further improve survival rates of pancreatic cancer patients. To that end, it is still highly important to advance our knowledge in pancreatic cancer biology and tumor stroma interactions. As a consequence, subgroups of patients will be identified and individualized therapies can be provided. In this review, metabolic reprogramming in pancreatic cancer focusing on lipid metabolism are discussed. Furthermore, we summarize several preclinical studies and clinical trials, also from other cancer types which may be important for future research and therapy of pancreatic cancer.

## 2. Fatty Acid Synthesis in Mammalian Cells and Its Critical Role in Pancreatic Cancer Cells

Metabolic reprogramming is now firmly recognized as a hallmark of cancer [4]. Cancer cells share a common phenotype of uncontrolled rapid cell growth and division. To be able to maintain cell proliferation, cancer cells need to keep generating cellular components such as DNA, proteins, and lipids. Activated lipid synthesis is essential for cancer cells, because lipids such as phospholipid bilayers are fundamental structural components enabling cell division. In mice, high fat diet (HFD) can increase oncogenic KRAS activity leading to more fibrotic stroma and enhanced PDAC development [5]. Lipids can sufficiently stimulate proliferation of pancreatic cancer cells lines [6], but a wide variety of tumors have activated de novo synthesis of fatty acids (FAs) irrespective of the levels of circulating lipids. In contrast to normal cells, cancer cells gain more than 93% of triacylglycerol FAs via de novo synthesis, which is activated by several signaling pathways [7]. Lipogenic enzymes are frequently overexpressed in many cancer types, including pancreatic cancer [8].

**Figure 1:**
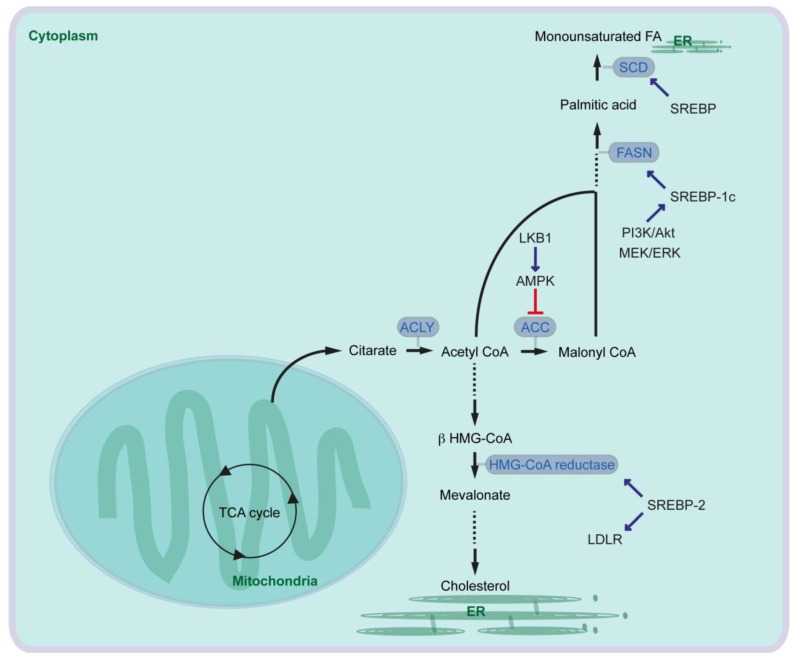
Central pathway for fatty acid and cholesterol synthesis (ACC: Acetyl-CoA carboxylase; ACLY: ATP-citrate lyase; AMPK: AMP-activated protein kinase; ER: endoplasmic reticulum; FA: fatty acid; FASN: fatty acid synthase; HMG: 3-hydroxy-3-methylglutaryl-coenzyme A; LKB1: liver kinase B1; LDLR: low-density lipoprotein receptor; SCD: ∆^9^-stearoyl-CoA desaturase; SREBP: sterol regulatory element-binding protein; TCA cycle: tricarboxylic acid cycle).

In the first-step of FA synthesis, cytoplasmic Acetyl-CoA is generated from citrate by the enzyme called ATP-citrate lyase (ACLY) and then converted to malonyl-CoA by Acetyl-CoA carboxylase (ACC). Acetyl-CoA and malonyl-CoA are coupled to the Acyl-carrier protein (ACP) domain of the multi-enzyme protein called fatty acid synthase (FASN), and via several reactions of acetyl-group condensation by the FASN in NADPH-dependent manner, a basic 16-carbon saturated FA palmitic acid is generated [9] (Figure 1).

In cancer cells, ACLY expression is markedly increased [7]. Glucose supports de novo lipid synthesis regulated by the PI3K/Akt signaling pathway, and knockdown of ACLY leads to impairment of glucose-dependent lipid synthesis and cell proliferation [10]. ACLY inhibition also reduces proliferation of several cancer cells. Furthermore, ACLY inhibition leads to reduced tumor growth in mice carrying xenografts of primary mouse PDAC lines generated from oncogenic KRAS with or without p53-mutation [11]. Similarly, ACC is also overexpressed in cancers [7], and inhibition of ACC can suppress FA synthesis and tumor growth in lung cancer mouse models where tumors are induced by oncogenic KRAS combined with *Trp53*-knockout, or by oncogenic KRAS combined with *Stk11*-knockout [12]. Serine/threonine kinase 11 (STK11), also known as liver kinase B1 (LKB1), activates AMP-activated protein kinase (AMPK). AMPK phosphorylates and inhibits ACCs (Figure 1).

It has been shown that serum FASN levels are higher in patients with PDAC, in patients with intraductal papillary mucinous neoplasia (IPMN) of the pancreas, and in patients with chronic pancreatitis than in health controls [13]. Pancreatic cancer patients with high expression of FASN show a shorter overall survival than patients with low FASN expression [14], and FASN expression is correlated with poor response to gemcitabine therapy in pancreatic cancer cells [14,15]. Expression of *FASN* is regulated by the transcription factor sterol regulatory element-binding protein 1c (SREBP1c) that is downstream of several signaling pathways and factors such as PI3K/Akt and MEK/ERK pathways (Figure 1). EGFR signaling is required for oncogenic KRAS-induced pancreatic tumorigenesis [16,17], and EGFR signaling activation also induces upregulation of FASN in pancreatic cancer cells in an ERK-dependent manner [18]. Along this line, PDAC patients with high SREBP1 expression have a shorter overall survival than patients with low SREBP1 expression, and knockdown of SREBP1 decreases pancreatic cancer cell viability and proliferation [19]. Taken together, oncogenic signaling pathways activate expression of lipogenic enzymes leading to aberrant activation of FA synthesis, which supports cancer cell development including pancreatic cancer.

## 3. Role of Saturated Fatty Acid and Fatty Acid Desaturase in Pancreatic Cancer

Pancreatic cancer risk is related to the intake of total fat, but especially of saturated and monounsaturated fatty acids (MUFAs) [20]. On the contrary, greater intake of omega-3 (ω-3 or n-3, contains double bond at the third carbon atom from the end of chain) polyunsaturated fatty acids (n-3 PUFAs), such as 18-carbon linolenic acid (ALA), 20-carbon eicosapentaenoic acid (EPA), and 22-carbon docosahexaenoic acid (DHA) reduces pancreatic cancer risk [21]. Consistently, mice fed with n-3 PUFAs exhibit decreased viability of pancreatic cancer cells in a xenotransplantation model, while saturated FA feeding stimulates tumor viability [22]. Arachidonic acid is also a type of PUFA, but the n-6 PUFA stimulates the growth of cyclooxygenase (COX) 2-positive pancreatic cancer cells [23], suggesting that n-3 and n-6 PUFAs have opposing effects for pancreatic cancer growth. In line with this, mice expressing n-3 fatty acid desaturase by transgene (called Fat-1) that catalyzes conversion of n-6 to n-3 FAs show attenuated oncogenic KRAS-mediated tumorigenesis without diet variation. Fat-1 expression leads to reduced COX-2 and anti-apoptotic Bcl-2 expression in KRAS-induced PDAC [24].

The desaturation of fatty acids occurs in the endoplasmic reticulum (ER) membranes. In mammalian cells, three types of fatty acid desaturases introduce carbon double bond at ∆^5^ (∆^5^-eicosatrienoyl-CoA desaturase, D5D), ∆^6^ (∆^6^-oleoyl(linolenoyl)-CoA desaturase, D6D) or ∆^9^ (∆^9^-stearoyl-CoA desaturase) (SCD) (“∆^x^” indicates carbon double-bond position counting from the carboxylic acid end). SCD is the rate-limiting enzyme catalyzing the synthesis of 16-carbon palmitoleate and oleate from palmitoyl-CoA and stearoyl-CoA. The expression of SCD is controlled by SREBP [25] (Figure 1). Expression of SCD1 is associated with tumor promotion, shorter survival of lung cancer patients (e.g., [26]) and with sorafenib resistance in liver cancer patients (e.g., [27]). Increased SCD1 expression is also observed in pancreatic cancer cells, and in pancreatic cancer patients [19,28].

## 4. Cholesterol Synthesis and LDL Synthesis in Pancreatic Cancer

Cholesterol is an essential structural component of cell membranes together with various phospholipids, sphingomyelin and glycolipids. Cholesterol is de novo synthesized from cytoplasmic acetyl-CoA through the mevalonate pathway. The rate-limiting step of the pathway is the conversion of 3-hydroxy-3-methylglutaryl-coenzyme A (HMG-CoA) to mevalonate by HMG-CoA reductase [29] (Figure 1). In addition to the mevalonate pathway, cells can increase their cholesterol contents thought receptor-mediated endocytosis of low-density lipoproteins (LDLs). The LDL receptor (LDLR) and HMG-CoA reductase are both transcriptional targets of SREBP-2 [30] (Figure 1). Expression of HMG-CoA reductase and LDLR is elevated in PDAC in an oncogenic KRAS mouse model [31]. It has been suggested that cholesterol intake is associated with the risk of pancreatic cancer [32]. Activation of the mevalonate pathway or aberrant cellular cholesterol intake via LDLR has also been associated with pancreatic cancer development. To that end, inhibition of HMG-CoA reductase leads to attenuation of pancreatic cancer cell proliferation [33]. Furthermore, LDLR silencing reduces ERK signaling activity and proliferation of PDAC cells, enhances response to gemcitabine chemotherapy. Increased expression of *Ldlr* gene has no significant effect on overall survival of pancreatic cancer patients, but high *Ldlr* expression is associated with an increased risk of tumor recurrence [31].

## 5. Roman Roads to Activate Lipid Metabolism in Pancreatic Cancer

**Figure 2:**
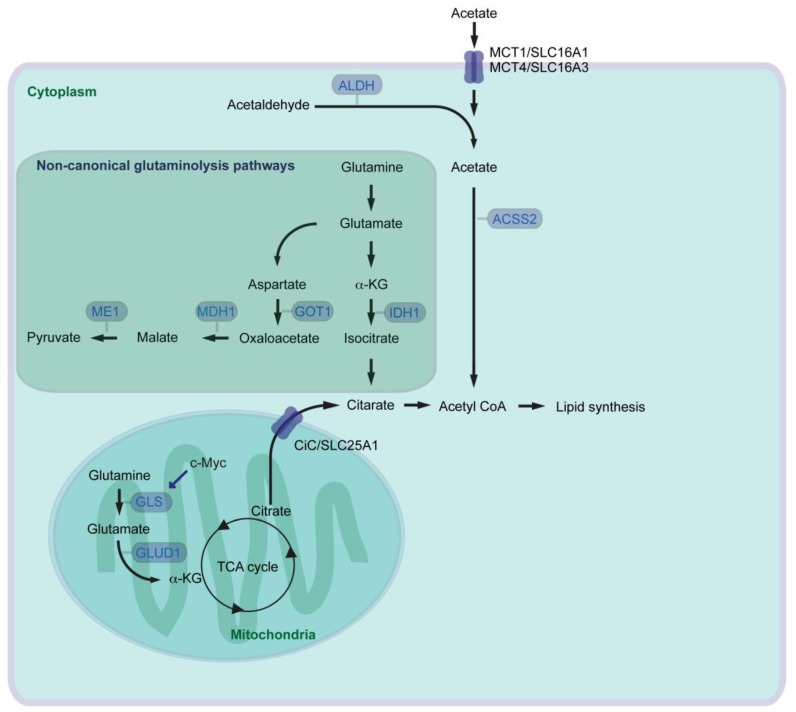
Canonical as well as non-canonical glutaminolysis pathways, and acetate pathway for supporting lipid metabolism in cancer (ACSS: acyl-CoA synthetase short chain family member; ALDH: Aldehyde dehydrogenase; CiC: citrate carrier; GLS: glutaminase; GLUD: glutamate dehydrogenase; GOT: aspartate transaminase; α-KG: α-ketoglutarate; IDH: isocitrate dehydrogenase; MCT: monocarboxylate transporter; MDH: malate dehydrogenase; ME: malate enzyme; SLC: solute carrier; TCA: tricarboxylic acid cycle).

By modulating activity of several metabolic pathways including glutamine and acetate metabolism, cancer cells aim for continuous generation of FAs necessary for cell growth. Glutamine is the most abundant amino acid in the blood. Next to glucose, glutamine serves as a carbon source for energy production and anabolic processes. In the canonical route of mitochondrial glutamine catabolism (glutaminolysis), glutaminase (GLS) catalyzes glutamine to glutamate. Glutamate is further converted to α-ketoglutarate (α-KG) by glutamate dehydrogenase (GLUD1), and α-KG can then enter the tricarboxylic acid cycle (TCA cycle) (Figure 2). Glutamine has been known since long time to be an essential nutrient for the proliferation of human cancer cells [34], and several oncogenes which activate glutaminolysis have been identified. Oncogenic c-Myc enhances expression of mitochondrial GLS supporting canonical glutaminolysis [35]. Myc-induced tumor development can be attenuated by heterozygous knockout of *Gls* leading to prolonged survival of mice [36]. A high rate of glutaminolysis can be beneficial for cancer cells, because it provides NADPH which is required for lipid biosynthesis [37]. Oncogenic KRAS also activates glutaminolysis but in a non-canonical way. Pancreatic cancer cells rely on a cytoplasmic glutaminolysis pathway producing pyruvate, which requires aspartate transaminase (GOT1, catalyzes aspartate to oxaloacetate), malate dehydrogenase (MDH1, catalyzes malate/oxaloacetate), and malate enzyme (ME1, catalyzes malate/pyruvate) (Figure 2) [38]. Consistently, inhibition of MDH1 activity leads to suppression of glutamine metabolism and reduction of pancreatic cancer cell growth [39]. With reprogramming of glutamine metabolism from the mitochondrial to the cytoplasmic system, cancer cells are able to keep FA synthesis intact: glutamine contributes to lipogenesis and cell growth in cancer cells via another non-canonical cytoplasmic glutaminolysis pathway with isocitrate dehydrogenase (IDH1, catalyzes α-KG/isocitrate) under hypoxia, or even with defective mitochondria [40,41,42].

Pyruvate generated via non-canonical glutaminolysis, or via glycolysis in general, can be transported into mitochondria and converted into acetyl-CoA by the pyruvate dehydrogenase complex. Mitochondrial acetyl-CoA is converted into citrate by the enzyme called citrate synthase. Pancreatic cancer show higher enzymatic activity of citrate synthase than control specimens taken from the adjacent pancreatic tissue [43]. Citrate is then transported from the mitochondria to the cytosol by the citrate carrier (CiC, SLC25A1) and converted into acetyl-CoA via ACLY as mentioned above (Figure 2). Acetyl-CoA is one of central regulators for multiple cellular processes, because acetyl-CoA is the source and precursor for both FA and cholesterol synthesis, and acetyl-CoA is a key determinant of protein/histone acetylation for regulating gene expression [44,45]. Otto Warburg has shown that many cancer cells preferentially convert pyruvate into lactate rather than transporting pyruvate into mitochondria and convert it into acetyl-CoA (“Warburg effect” [46]). Alternative source of acetyl-CoA could therefore be necessary for sufficiently supporting lipid synthesis and cancer cell growth, especially under hypoxic conditions. There are 26 acyl-CoA synthetases (ACS) identified in the human genome. Among those, three enzymes are capable of catalyzing synthesis of acetyl-CoA from acetate in an ATP-dependent manner [47]. The short chain ACS (ACSS) family (acetyl-CoA synthetase) ACSS1 and ACSS3 are mitochondrial enzymes, and ACSS2 localizes to both the cytoplasmic and nuclear compartments. Silencing of *ACSS2* in cancer cells reduces incorporation of acetyl unit from acetate into either lipids or histones more than suppression of *ACSS1* or *ACSS3*. ACSS2 is highly expressed in several human tumors, and loss of ACSS2 suppresses tumor development in a mouse cancer model of cMyc combined with PTEN knockout [48]. Under metabolic stress such as hypoxia and/or low-nutrition conditions, expression of ACSS2 is elevated and ACSS2 promotes acetate uptake for lipid synthesis and membrane phospholipids in several cancers including pancreatic cancer cells [49,50]. Oxygen and serum restriction promotes nuclear localization of ACSS2, recaptures de novo synthesized acetate, and maintains histone acetylation [50].

The major dietary sources of acetates are acetate-containing foods such as processed meats, ethanol for oxidative catabolism and indigestible carbohydrates [51]. The transporters involved in uptake of acetate in cancer cells remain largely unexplored. Monocarboxylate transporter 1 and 4 (MCT1, MCT4) are the solute carrier (SLC) group of membrane transport proteins, which are expressed in many types of cancer [51]. MCT1 and MCT4, also known as SLC16A1 and SLC16A3 respectively, are generally involved in transport of lactate, pyruvate and ketone bodies. It has been suggested that MCT1 and MCT4 are also involved in acetate transport [52] (Figure 2). MCT4 expression is regulated by the PI3K/Akt signaling pathway and an important regulator of cancer cell survival [53]. In line with this, shRNA-mediated knockdown of MCT4 results in attenuated development of pancreatic cancer cell xenograft in mice. Furthermore, pancreatic cancer patients with high MCT4 expression have shorter survival after resection [54].

Acetate can also be de novo synthesized from acetaldehyde by aldehyde dehydrogenase (ALDH). ALDHs catalyze the oxidation of broad spectrum of aldehyde molecules such as acetaldehyde, retinaldehyde, and aldehydes associated with amino acid metabolism [55]. There has been 19 ALDHs identified in human, among those, mitochondrial ALDH2 isozyme predominantly catalyzes the oxidation of acetaldehyde [56]. ALDH1A members (ALDH1A1, ALDH1A2, and ALDH1A3) are located mainly in the cytoplasm and primarily oxidize retinaldehyde into retinoic acid (RA), but are also involved in oxidizing acetaldehyde [55,56]. Enriched expression of ALDH1A3 has been observed in many developmentally unrelated tumors including pancreatic cancer [57]. ALDH1A3 labels an aggressive subtype of human PDAC, and patients with ALDH1A3 expression have shorter survival after surgical resection [58], and inhibition of ALDH1A3 can lead to reduction of cancer cell growth [59]. It is still not known whether ALDHs are just aldehyde-oxidizing enzymes, or have also additional functions supporting cancer development. Irrespective, elevated expression of ALDH can support de novo acetate synthesis, and acetate can be further used for acetyl-CoA and lipid synthesis.

## 6. Pancreatic Stellate Cells and Lipid Metabolism in Pancreatic Cancer

One of the characteristic features of PDAC is abundant desmoplastic reaction with prominent stromal compartments. Tumor stroma comprises heterogeneous cellular and non-cellular components such as fibroblasts, blood vessels, immune cells, pancreatic stellate cells (PSCs), and the extracellular matrix (ECM). Stroma compartments can make up over 90% of the tumor mass, which drives the hypoxic tumor microenvironment [1]. Dense tumor stroma can be a physical and metabolic barrier leading to massive reduction of therapeutic efficacy. ECM proteins are produced by activated PSCs supporting pancreatic fibrogenesis. In the healthy pancreas, PSCs are in the quiescent state and retain vitamin A-containing lipid droplets. Extracellular signals such as TGF-β, TNF-α, and other cytokines and growth factors, stress, acetaldehyde, ethanol and other factors modulate PSC activation [60]. Albumin may inactivate PSCs by maintaining formation of vitamin A droplets, and downregulation of albumin by TGF-β leads to PSC activation due to loss of vitamin A [61].

Activated PSCs produce several stimulators supporting angiogenesis and cancer cell proliferation [60]. In addition, PSCs support PDAC by providing metabolites which are required for PDAC development. Autophagy-dependently generated alanine from PSCs is taken-up by PDAC cells, who utilize it for fatty acid biosynthesis. PDAC cells stimulate autophagy in PSCs to be able to consume released alanine [62]. In line with this, oncogenic KRAS in PDAC regulates not only cell-autonomous signaling in pancreatic cancer cells, but also leads to production of factors activating PSCs in a cell non-autonomous manner. Activated PSCs produce reciprocal signals back to pancreatic cancer cells [63]. Lipids can also be tumor-stroma communication mediators for cancer and stroma cells. High fat diet-fed mice with oncogenic KRAS and p53 mutation-induced pancreatic tumors (KPC mice) show larger primary PDACs and higher rates of metastasis than mice fed with standard diet. Fatty acids presumably generated by adipose tissue are incorporated into pancreatic cancer cells, and increases LD formation and tumor cell migration [64]. Inactivation of PSCs prevents pancreatic tumor growth and increases the response to chemotherapy in KPC mice with high fat diet [65]. Vitamin D receptor activation of PSCs attenuates inflammation and fibrosis, and enhances gemcitabine chemotherapy response [66].

## 7. Hypoxic Environment in Stroma Drives Angiogenesis, Tumorigenesis, and Lipid Metabolism

The hypoxic microenvironment leads to activation of specific molecular programs to counteract the hypoxic challenge, where hypoxia inducible factors (HIFs) play a central role. HIFs are composed of an O_2_-regulated α subunit (e.g., HIF-1α) and a constitutively expressed β subunit (e.g., HIF1-β), also called aryl hydrocarbon receptor nuclear translocator (ARNT). Under normoxic conditions, the synthesized HIF-1α subunit is subjected to hydroxylation on proline residues by prolyl hydroxylase domain (PHD) proteins, where O_2_ and α-KG are used as substrates. Hydroxylated HIF-1α is further subjected to modification, ubiquitination marking for proteasomal degradation. Under hypoxic conditions, the hydroxylation reactions are inhibited and stabilized HIF-1α can dimerize with HIF-1β. The resulting HIF-1 heterodimer activates HIF1-dependent target gene expression [67]. The PI3K/Akt/mTOR, ERK, and NF-κB pathways also regulate HIF-1α, which can lead to HIF activation even in a normoxic condition [68,69]. Elevated levels of tumor HIF-1α are associated with decreased pancreatic cancer patient survival [70].

The HIF-1 transcription factor complex regulates expression of a variety of target genes, which are involved in e.g., angiogenesis, metastasis and metabolism [69]. Vascular endothelial growth factor (VEGF), a HIF-1 target, is a key regulator of neovascularization [71]. Pancreatic cancer patients with elevated preoperative serum VEGF levels have poor overall survival [72]. VEGF can activate several signaling pathways such as RAS/RAF/ERK and PI3K/Akt pathways [73], which may further regulate lipid synthesis. It has been suggested that hypoxia upregulates *FASN* gene expression thorough SREBP-1 [74]. Hypoxia induced HIF-1 regulates also glutamine metabolism and supports lipid synthesis. HIF-1 activation reduces the activity of α-ketoglutarate dehydrogenase (α-KGDH, catalyzes α-KG/succinate) which drives the metabolic shift from TCA cycle to IDH-mediated FA synthesis (non-canonical glutaminolysis pathway, see above) [75]. HIF-1α further downregulates medium- and long-chain acyl-CoA dehydrogenase (MCAD and LCAD) leading to reduced lipolysis/β-oxidation and cancer progression [76]. It has also been suggested that hypoxia induces HIF-1α-dependent accumulation of lipid droplets in tumor cell lines, where de novo FA synthesis is repressed [77].

## 8. Role of Lipid Droplets in Cancer

Hypoxia regulates lipid uptake and lipid droplet (LD) formation. LDs have been increasingly recognized as important regulators in metabolic diseases and cancer. LDs are dynamic intracellular organelles that store neutral lipids in cells, including triacylglycerol (TAG) and cholesterol ester [78]. The basic structure of LDs is a mass of lipid esters covered by a phospholipid monolayer [79], making LDs separated from the aqueous cytosol [80]. In mammalian cells, acyl-CoA diacylglycerol acyltransferase (DGAT) enzymes, DGAT1 and DGAT2, synthesize TAG, and acyl-CoA cholesterol acyltransferase (ACAT) enzymes, ACAT1 and ACAT2, generate sterol esters (SEs). Neutral lipid synthesis enzymes are primarily localized in the ER, but also found in LDs except DGAT2 [80]. The accumulation of cholesteryl ester by loss of PTEN and activation of PI3K/Akt signaling is associated with cancer aggressiveness. Depletion of ACAT1 by shRNA reduces cancer proliferation and impairs cancer invasion [81]. Aberrant accumulation of cholesteryl ester is also observed in pancreatic cancer patients, and ACAT1 expression is correlated with poor patient survival. Inhibition of ACAT1 or knockdown with shRNA activates ER stress response/ unfolded protein response (UPR) signaling and reduces pancreatic cancer cell proliferation as well as tumor development in mice [82].

A number of studies have identified and characterized many LD-associated proteins, including structural proteins and enzymes involved in cholesterol and FA metabolism, and proteins regulating membrane trafficking [78]. Furthermore, numerous proteins associated with LDs act as gatekeepers as well as messengers interacting with cytoplasmic proteins [83]. The perilipin family member of proteins includes perilipin (PLIN1), adipose differentiation-related protein (adipophilin, ADRP, PLIN2), tail-interacting protein of 47 kDa (TIP47, PLIN3), S3-12 (PLIN4), and OXPAT/MLDP/LSDP5 (PLIN5) [78,79]. Each PLIN protein exhibits individual expression pattern during tumorigenesis, for example PLIN2 expression correlates with tumor cell proliferation [84]. HIF2α/PLIN2 promotes lipid storage and activity of HIF2α is correlated with *PLIN2* gene expression. PLIN2 knockdown activates UPR signaling and attenuates tumor growth in clear-cell renal carcinoma [85]. The role of PLIN proteins in the tumor microenvironment of pancreatic cancer has also been analyzed. Pigment epithelium-derived factor (PEDF) is a potent antiangiogenic factor and more than half of pancreatic cancers have reduced levels of PEDF expression. Oncogenic KRAS expression together with PEDF-deficiency in mice increases peripancreatic fat with adipocyte hypertrophy and intrapancreatic infiltration of adipocytes. The stroma of the mice with oncogenic KRAS and PEDF-deficiency demonstrates elevated expression PLIN2 and PLIN3, which is associated with increased adipogenesis [86]. Cell death-inducing DFF45-like effector (CIDE) family proteins such as Cidea, Cideb, and Fat-specific protein 27 (FSP27, Cidec) are also known as LD- and ER-associated proteins, which play roles in controlling lipid storage and regulate insulin sensitivity [87]. It has so far not been analyzed whether CIDE proteins have critical roles in (pancreatic) cancer development.

## 9. Targeting Lipid Metabolism and Therapy Options for Cancer Patients

**Figure 3:**
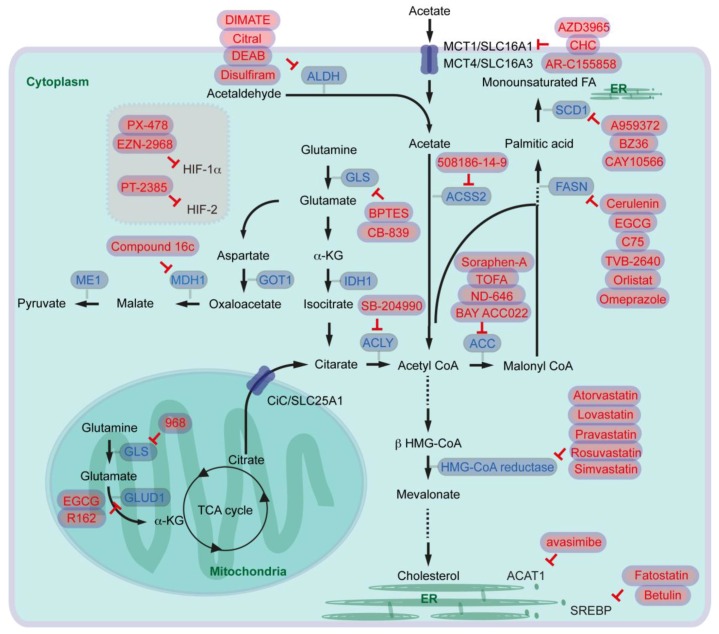
Overview of inhibitors for targeting lipid metabolism in cancers (ACAT: acyl-CoA cholesterol acyltransferase; ACC: Acetyl-CoA carboxylase; ACLY: ATP-citrate lyase; ACSS: acyl-CoA synthetase short chain family member; AMPK: AMP-activated protein kinase; ALDH: Aldehyde dehydrogenase; CiC: citrate carrier; ER: endoplasmic reticulum; FA: fatty acid; FASN: fatty acid synthase; GLS: glutaminase; GLUD: glutamate dehydrogenase; GOT: aspartate transaminase; HIF: hypoxia inducible factor; HMG: 3-hydroxy-3-methylglutaryl-coenzyme A; IDH: isocitrate dehydrogenase; α-KG: α-ketoglutarate; LKB1: liver kinase B1; LDLR: low-density lipoprotein receptor; MCT: monocarboxylate transporter; MDH: malate dehydrogenase; ME: malate enzyme; SCD: ∆^9^-stearoyl-CoA desaturase; SLC: solute carrier; SREBP: sterol regulatory element-binding protein; TCA: tricarboxylic acid cycle).

It has now been increasingly accepted that targeting or modulating lipid metabolism in cancer cells as well as in stroma is an emerging strategy combating against cancer. To that end several inhibitors/drugs have been developed and tested in several preclinical and clinical trials (or trials are ongoing). In this chapter, several drugs/inhibitors modulating lipid metabolism not only in pancreatic cancer, but also in other cancer types potentially relevant for future pancreatic cancer care are discussed (see Figure 3 and Table 1). Concerning adverse effects or contraindications please check further reviews specialized for each drug.

### 9.1. Targeting Fatty Acid Synthesis Pathway and Lipid Desaturation

For targeting fatty acid synthesis, several inhibitors for ACLY, ACC, and FASN blockade have been generated. SB-204990 has been proposed as an ACLY inhibitor. In a preclinical study, it has been shown that intraperitoneally administered SB-204990 reduces tumor xerograph development of murine primary pancreatic cancer cells in mice [11]. For inhibiting ACC, Soraphen-A and TOFA have been tested in several cancer cell culture models [88,89]. In a preclinical mouse xenograph model, intraperitoneally administered TOFA reduces human ovarian cancer cell development [90]. Furthermore, oral administration of another ACC inhibitor ND-646 has been described to attenuate tumor growth of lung cancer cells in mice [12]. For pancreatic cancer, BAY ACC022 has been tested by oral administration: after inoculation of pancreatic cancer cells mice administered with the ACC inhibitor had less tumor volume than controls [91].

Targeting FASN can be performed by several different inhibitors, since FASN is a multi-enzyme protein complex. Mammalian FASN consists of two identical polypeptides. Each polypeptide includes seven catalytic domains namely, ACP, malonyl/acetyltransferase (MAT), β-ketoacyl-ACP synthase, β-ketoacyl-ACP reductase, 3-hydroxyacyl-ACP dehydrase, enoyl-CoA reductase, and palmitoyl-ACP thioesterase. Cerulenin, Epigallocatechin-3 gallate (EGCG), and C75 block β-ketoacyl-ACP synthase-domain of FASN. TVB-2640 inhibits β-ketoacyl-ACP reductase. The β-lactone orlistat blocks thioesterase-domain, and enoyl-CoA reductase-domain can be blocked by triclosan [7,92]. It has been shown in a mouse model that intraperitoneally administered cerulenin suppresses liver metastasis of colon cancer cells in mice [93]. Blockade of FASN with EGCG has been considered for broad range of cancer types such as prostate, lung, breast, and colorectal cancer, where several phase 2 and phase 3 clinical trials are ongoing. For pancreatic cancer, it has been shown that EGCG inhibits growth of tumors orthotopically implanted in mice [94]. TVB-2640 has entered clinical trials, e.g., for people with resectable colon cancer (phase 1), for advanced breast cancer (phase 2), or for high grade astrocytoma (phase 2). Orlistat is a US food and Drug Administration (FDA)-approved anti-obesity drug, and it has been shown that orlistat reduces human pancreatic cancer cell growth [14,95]. As orlistat has low oral bioavailability which may reduce cancer therapy effect [7], several studies suggest combination therapies with ALCAR (AMPK activator) for prostate cancer [96], or simultaneous targeting glycolysis, glutaminolysis, and FA synthesis by lonidamine, 6-diazo-5-oxo-l-norleucine which are combined with orlistat for colon cancer therapy [97]. Alternatively, other inhibitors of thioesterase-domain can be identified via in silico screening of FDA-approved drugs. Lansoprazole, rabeprazole, omeprazole, and pantoprazole are proton pump inhibitors, but also function as inhibitors of thioesterase activity, which can induce pancreatic cancer cell death [98]. Among these drugs, omeprazole has been entered a clinical trial as a FASN inhibitor to test efficacy during neoadjuvant chemotherapy for breast cancer patients (phase 2).

Targeting lipid desaturase SCD1 has also been considered for cancer therapy. An inhibitor called A939572 has been applied for renal cell carcinoma treatment. Oral administration of A939572, which is re-suspended in water, inhibits the development of tumor xenografts in mice [99]. Intraperitoneal injection with another SCD1 inhibitor called BZ36 reduces prostate cancer xenografts in mice [100]. Furthermore, it has been shown that pretreatment with CAY10566 (also an SCD1 inhibitor) suppresses ovarian tumor growth after inoculation of cancer stem cells, where inhibition of SCD1 impairs cancer cell stemness [101]. So far SCD1 has not been entered preclinical studies for pancreatic cancer therapy.

### 9.2. Targeting Cholesterol Synthesis and SREBP

Inhibiting de novo cholesterol synthesis by blockage of the rate-limiting enzyme HMG-CoA reductase has also been considered for cancer therapy. Several statin derivatives such as atorvastatin, lovastatin, pravastatin, rosuvastatin, simvastatin have entered clinical trials. Among the derivatives, atorvastatin and simvastatin have been considered for pancreatic cancer treatment. One clinical study performs metabolic treatment with metformin, atorvastatin, doxycycline, and mebendazole for several types of cancer patients including pancreatic cancer (phase 3). A phase 2 study with gemcitabine alone, or gemcitabine combined with simvastatin has been conducted for advanced pancreatic cancer patients.

As a key regulator of expression of FASN and other enzymes in fatty acid synthesis, SCD, LDLR, and HMG-CoA reductase, SREBP1c and SREBP2 are potential targets for cancer therapy, and betulin and fatostatin have been proposed as SREBP inhibitors. Betulin has initially been shown to improve hyperlipidemia and insulin resistance, and to reduce atherosclerotic plaques [102]. Intraperitoneal injection of betulinic acid combined with mithramycin A blocks the development of pancreatic cancer xenografts in mice [103]. Another SREBP inhibitor fatostatin has been tested for glioblastoma cell xenografts, there intraperitoneal treatment with fatostatin reduced xenograft growth in mice [104].

### 9.3. Targeting Glutamine and Acetate Metabolisms

As mentioned above, cancer cells can reprogram glutamine metabolism supporting continuous de novo FA synthesis, and several studies are trying to target this pathway. For inhibiting GLS glutamate synthesis, drugs called 968, BPTES, and CB-839 have been proposed [105,106]. It has been suggested that 968 inhibits mitochondrial GLS and blocks growth and invasiveness of cancer cells [107]. Intraperitoneal administration of BPTES reduces glutamate levels specifically in Myc-induced liver cancer (but not in the normal liver), leading to prolonged survival of mice [36]. BPTES blocks lymphoma cell proliferation also under hypoxic conditions, and intraperitoneal injection attenuates lymphoma cell xenografts in mice [108]. Intravenous injection of BPTES nanoparticles reduces pancreatic cancer xenografts in mice, and combination with intraperitoneal injection of metformin enhances therapeutic effects [109]. Another GLS inhibitor CB-839 has already been considered in several clinical studies. This drug has entered also phase 2 clinical trials for studying its effect in a broad range of cancer types, such as clear cell renal carcinoma, breast cancer, and colorectal cancer. CB-839 has exhibited also anti-proliferative activity in pancreatic cancer cells. However, oral gavage of CB-839 has no antitumor activity in mice with oncogenic KRAS combined with *Trp53*-knockout. In addition, the mice treated with CB-839 show marginally shorter survival than the group without CB-839 treatment [110]. Further investigations are therefore required to judge whether GLS inhibition is a potential therapeutic option for pancreatic cancer patients.

As GLUD1 inhibitors EGCG and R162 have been considered [105]. Treatment with R162 inhibits proliferation of several cancer cells including primary leukemia cells isolated from patients. Furthermore, intraperitoneal injection of R162 inhibits the development of lung cell tumor xenografts in mice [111]. Another inhibitor EGCG is known as FASN β-ketoacyl-ACP synthase-domain inhibitor (see above). EGCG is but also widely recognized as a GLUD1 inhibitor, and EGCG by intraperitoneal administration inhibits growth of Myc-mediated neuroblastoma cell xenografts in mice [112]. Enzymes involved in non-canonical glutaminolysis pathway can also be targeted. Methyl 3-(3-(4-(2,4,4-trimethylpentan-2-yl)phenoxy)-propanamido)benzoate (named compound 16c) has been synthesized as a MDH inhibitor. This inhibitor blocks both cytoplasmic MDH1 and mitochondrial MDH2 enzymes. It has been shown that intraperitoneal administration of this inhibitor attenuates the development of colon cancer cell xenografts [113]. Further evaluations of MDH inhibitors, together with the development of specific GOT1, ME1 inhibitors are expected as therapeutic options in pancreatic cancer [114].

Targeting acetate metabolism is also an emerging concept as a therapy for cancer patients and there are several ACSS inhibitors for blocking acetyl-CoA synthesis. One example is allicin, which is a naturally occurring product from garlic [115]. Another compound 1-(2,3-di(thiophen-2-yl)quinoxalin-6-yl)-3-(2-methoxyethyl)urea (PubChem CID: 2300455; here referred to as 508186-14-9) was identified as a ACSS2-specific inhibitor via high-throughput screening [48]. Since knockout or siRNA knockdown of Acss2 inhibits cancer cell proliferation, direct evidence that ACSS2-specific inhibitors blocks cancer cell development in vitro and in vivo, is expected.

Blocking acetate import for cancer cells is another strategy. One possibility is inhibiting transporters involved in uptake of metabolites including acetate. AZD3965, a-cyano-4-hydrocinnamate (CHC), and AR-C155858 are examples of MCT1 inhibitors [116]. AZD3965 has already entered a phase 1 clinical trial in patients with advanced cancers such as prostate cancer, gastric cancer, and diffuse large B cell lymphoma. CHC has been suggested to impair glioblastoma cell proliferation, migration, and survival [117]. AR-C155858 blocks proliferation of gastric cancer cells with high MCT expression in vitro and in vivo in an intraperitoneal injection model in mice [118]. In most of the studies MCT1 inhibitors are considered to block lactate uptake. How much impact acetate uptake alone (beside lactate uptake) for tumor development has, needs to be evaluated.

Another possibility to provide acetate for cancer cells is de novo synthesis catalyzed by ALDH. Several ALDH inhibitors have been proposed and characterized which are discussed elsewhere in detail [119]. The major mitochondrial enzyme catalyzing acetaldehyde for producing acetate is ALDH2, but cytoplasmic ALDH1 can be involved in oxidizing acetaldehyde as well (see above). Here, we focus on several published studies as well as clinical trials with ALDH1A and/or ALDH2 inhibitors namely 4-amino-4-methyl-2-pentyne-1-al (AMPAL)/demethylampal thioester (DIMATE), citral, diethylaminobenzaldehyde (DEAB), and disulfiram. AMPAL was tested as an irreversible inhibitor of ALDH nearly three decades ago whereupon it inhibited proliferation of leukemia cell lines. Furthermore, administration of AMPAL increased survival of mice intraperitoneally grafted with leukemia cells [120]. However, AMPAL tends to polymerize due to the simultaneous presence of a primary amine and aldehyde group. To solve the drawback, an irreversible ALDH1 inhibitor DIMATE has been synthesized [121]. DIMATE induces apoptosis of chemoresistant mouse lymphoid cells that are also resistant to disulfiram. DIMATE is cytotoxic for leukemic stem cells, but not for healthy hematopoietic stem cells. Administration of DIMATE in mice decreases leukemia cells which are derived from acute myeloid leukemia patients and intravenously transplanted [122]. DIMATE also promotes cell death of melanoma cells and inhibits tumor growth in immunocompetent, immunosuppressed and patient-derived xenograft mouse models [123]. Citral inhibits ALDH1A3 and ALDH2. Citral is known to be degraded at acidic pH and under oxidative stress. Intraperitoneal injection of free citral is therefore ineffective at reducing tumor growth of breast cancer cells implanted in mice. Nanoparticle encapsulated citral (citral-NP) has been developed which can enhance in vivo bioavailability of the inhibitor. It has been shown that tail vein injection of citral-NP reduces tumor growth of ALDH1A3-overexpressing breast cancer cells implanted in mice [59]. Another inhibitor called DEAB is suggested to block ALDH1A1, ALDH1A3, and ALDH2 [59]. Treatment with DEAB increases sensitivity to doxorubicin and paclitaxel therapy in breast cancer cells [124]. In a preclinical study, DEAB also inhibits metastasis of implanted breast cancer cells to lung in mice [125]. Furthermore, DEAB and disulfiram re-sensitize lung cancer cells to cisplatin [126], and disulfiram also sensitizes breast cancer cells to paclitaxel and cisplatin [127]. Pancreatic cancer cells with high ALDH activity are more sensitive to disulfiram, and oral administration of disulfiram makes gemcitabine more effective in mice [128]. A phase 1 clinical trial has been initiated to treat pancreatic cancer patients in combination with gemcitabine chemotherapy (although here disulfiram is used as a potential inhibitor of muscle degradation). In addition, several phases 1 and 2 studies have been registered for neuroblastoma, breast and prostate cancer.

### 9.4. Activating Vitamin D Receptor Signaling, Targeting HIF and Proteins Associated with Lipid Droplet

VDR activation has been suggested to attenuate inflammation and fibrosis, enhances gemcitabine chemotherapy in the pancreas (see above), and it is a promising therapeutic option for pancreatic cancer. A phase 2 clinical trial has been registered for patients with pancreatic cancer, where the PD-1 inhibitor pembrolizumab with the vitamin D analog paricalcitol is used. Further, a clinical trial is considered for treating pancreatic cancer patients prior to surgery for resectable PDAC with gemcitabine, abraxane in combination with paricalcitol. In addition, phase 1 trials for metastatic breast cancer with cholecalciferol, colon cancer with vitamin D3, phase 2 trial for metastatic colorectal cancer with FOLFOX, bevacizumab, and vitamin D, phase 3 trial for melanoma patients with cholecalciferol have been registered.

The hypoxic microenvironment or activation of several signaling pathways leads to activation of HIF, which regulates angiogenesis, metastasis, metabolism, and pancreatic cancer development. A HIF-1α inhibitor PX-478 attenuates pancreatic cancer cell proliferation, and it supports the effects of arsenic trioxide treatment [129]. Combined treatment with gemcitabine and PX-478 enhances antitumor effects in pancreatic cancer xenografts in immune-competent mice [130]. A phase 1 trial has been conducted to determine the safety and biological activity for patients with advanced metastatic cancer. Another example of an HIF-1α inhibitor is EZN-2968, an RNA antagonist. EZN-2968 reduces HIF-1α expression as well as VEGF secretion, and attenuates tumor growth [131]. EZN-2968 (other name: SPC2968/RO7070179) has also been entered phase 1 clinical trials. Several phase 2 studies have been registered with the HIF-2 heterodimerization inhibitor PT2385 for patients with clear cell renal carcinoma and glioblastoma. Inhibition of ACAT1 has been considered for targeting lipid droplets. It has been shown that intragastric administration of the ACAT1 inhibitor avasimibe reduces melanoma and lung cancer tumor xenografts in mice, and mice treated with avasimibe survive longer than control mice after melanoma or lung cancer cell injection [132]. Intraperitoneal injection of avasimibe also reduces development of pancreatic cancer xenografts in mice [82].

## 10. Conclusions

From preclinical cancer models to clinical trials, it has now become apparent that lipid metabolism plays a crucial role in pancreatic cancer. Cancer cells reprogram a broad range of metabolic systems to support lipid synthesis and provide optimal condition for cancer development. Cancer cells activate not only signaling cascades and metabolic reprogramming in cancer cells in a cell-autonomous manner, but also modulate systems in stroma cells including stellate cells in a cell-non-autonomous way. Stroma cells can also produce reciprocal signals back to cancer cells. As dynamic organelles, lipid droplets are also involved in metabolism and cancer development. It is important to further increase our knowledge of lipid metabolism in pancreatic cancer cells. However, understanding the tumor microenvironment and stroma, and characterization of factors of intercellular communication are also necessary for developing novel strategies of the future therapy of pancreatic cancer.

## Figures and Tables

**Table 1:** Overview of completed and ongoing clinical trials with inhibitors blocking lipid metabolism-associated factors.

Inhibitor Name	Main Target	Stage of Clinical Trial	NCT Number	Addendum
EGCG	FASN	phase 2 (prostate cancer)	NCT00676780	-
EGCG	FASN	phase 2/3 (prostate cancer)	NCT01105338	-
EGCG	FASN	phase 2 (lung cancer)	NCT00573885	-
EGCG	FASN	phase 2 (breast cancer)	NCT02580279	-
EGCG	FASN	phase 2 (colorectal cancer)	NCT01360320	-
TVB-2640	FASN	phase 1 (colon cancer)	NCT02980029	-
Omeprazole	FASN	phase 2 (breast cancer)	NCT02595372	test efficacy neoadjuvant chemotherapy
Atorvastatin	HMG-CoA reductase	phase 3 (pancreatic cancer and other cancer types)	NCT02201381	combination treatment with metformin, doxycycline, and mebendazole
Simvastatin	HMG-CoA reductase	phase 2 (pancreatic cancer)	NCT00944463	combination treatment with gemcitabine
CB-839	GLS	phase 2 (clear cell renal cell carcinoma)	NCT03163667	combination treatment with everolimus
CB-839	GLS	phase 1/2 (clear cell renal cell carcinoma, melanoma, lung cancer)	NCT02771626	combination treatment with nivolumab
CB-839	GLS	phase 2 (breast cancer)	NCT03057600	combination treatment with paclitaxel (Pac-CB)
CB-839	GLS	phase 1/2 (solid tumor and fluoropyrimidine resistant PI3KCA mutant colorectal cancer)	NCT02861300	combination treatment with capecitabine
AZD3965	MCT1	phase 1 (solid tumor, prostate cancer, gastric cancer, lymphoma)	NCT01791595	-
Disulfiram	ALDH	phase 1 (pancreatic cancer)	NCT02671890	combination treatment with gemcitabine
Disulfiram	ALDH	phase 2 (breast cancer)	NCT03323346	-
Disulfiram	ALDH	phase 1 (prostate cancer)	NCT02963051	combination treatment with copper
Disulfiram	ALDH	phase 2 (glioblastoma)	NCT01777919	combination treatment with copper and temozolomide
Paricalcitol	VDR	phase 2 (pancreatic cancer)	NCT03331562	combination treatment with pembrolizumab
paricalcitol	VDR	pilot trial (pancreatic cancer)	NCT02030860	combination treatment with abraxane and gemcitabine
Cholecalciferol (vitamin D3)	VDR	phase 1 (breast cancer)	NCT02186015	-
Cholecalciferol (vitamin D3)	VDR	phase 3 (melanoma)	NCT01264874	-
Cholecalciferol (vitamin D3)	VDR	phase 1 (colon cancer)	NCT02172651	-
Vitamin D	VDR	phase 2 (metastatic colorectal cancer)	NCT01516216	combination treatment with FOLFOX and bevacizumab
PX-478	HIF-1α	phase 1 (solid tumor, lymphoma)	NCT00522652	-
EZN-2968, SPC2968, RO7070179	HIF-1α	phase 1 (hepatocellular carcinoma)	NCT02564614	-
EZN-2968, SPC2968, RO7070179	HIF-1α	phase 1 (solid tumor with liver metastasis)	NCT01120288	-
EZN-2968, SPC2968, RO7070179	HIF-1α	phase 1 (solid tumor or lymphoma)	NCT00466583	-
PT2385	HIF-2	phase 2 (glioblastoma)	NCT03216499	-
PT2385	HIF-2	phase 2 (clear cell renal cell carcinoma)	NCT03108066	-
